# Application of green synthesized WO_3_-poly glutamic acid nanobiocomposite for early stage biosensing of breast cancer using electrochemical approach

**DOI:** 10.1038/s41598-021-03209-8

**Published:** 2021-12-14

**Authors:** Hassan Nasrollahpour, Abdolhossein Naseri, Mohammad-Reza Rashidi, Balal Khalilzadeh

**Affiliations:** 1 Department of Analytical Chemistry, Faculty of Chemistry, University of Tabriz, PO Box 51644-14766, Tabriz, Iran; 2 Department of Medicinal Chemistry, Faculty of Pharmacy, Tabriz University of Medical Sciences, Tabriz, Iran; 3 Stem Cell Research Center, Tabriz University of Medical Sciences, 51664-14766 Tabriz, Iran

**Keywords:** Cancer, Biomarkers, Diseases, Medical research, Chemistry, Nanoscience and technology

## Abstract

Biopolymer films have drawn growing demand for their application in the point of care domain owing to their biocompatibility, eco-friendly, and eligibility for in vivo analyses. However, their poor conductivity restricts their sensitivity in diagnostics. For high-quality electrochemical biosensor monitoring, two vital factors to be greatly paid attention are the effective merge of amplification modifiers with transducing surface and the superior linking across the recognition interface. Here, we introduce an enzyme-free electrochemical biosensor based on electrosynthesized biocompatible WO_3_/poly glutamic acid nano-biocomposites to address the hardships specific to the analysis of circulating proteins clinical samples. In addition to its green synthesis route, the poor tendency of both components of the prepared nano-biocomposite to amine groups makes it excellent working in untreated biological samples with high contents of proteins. Several electrochemical and morphological investigations (SEM, EDX, and dot mapping) were fulfilled to gain a reliable and trustful standpoint of the framework. By using this nanobiosensor, the concentration of HER-2 was detectable as low as 1 fg mL^−1^ with a wide linear response between 1 ng mL^−1^ and 1 fg mL^−1^. Meanwhile, the protocol depicted ideal specificity, stability, and reproducibility for the detection of HER-2 protein in untreated serum samples of breast cancer patients.

## Introduction

Considering the worldwide concerns, breast cancer (BC) adapted a great diagnostic and prognostic importance. According to the WHO reports, BC is documented as the number one priority incidence among women (2.1 million individuals per year). Reports of 627,000 deaths in 2018 (15% of cancer-related mortality among women) exhibit the importance of early and precise detection of BC^1^. Therefore, the design of new methodologies for tracking breast cancer biomarkers is timely and significant^2,3^. Among many diagnostic ways, circulating tumor markers (CTMs) have attracted much attention currently. These markers are released from tumor cells into the bloodstream. This leads their concentration to be raise in blood as level as the cancer progression^4,5^. As a simulative protein factor, human epidermal growth factor receptor 2 (HER-2) is found in 20–30% of breast cancer incidences. The basal level of HER-2 in healthy individuals is estimated as about ≤ 15 ng mL^−1^ (physiologic < 15 ng mL^−1^ < pathologic). During anaplastic changes and tumor development, the level of HER-2 protein is increased in the blood of patients^6–8^. Therefore, the cancer incidence can be detected via comparing the analytical signals for cancerous samples with the signals for healthy samples. This emphasizes the appeal for highly sensitive and efficient probing tools. The screening of cancer-related circulating proteins, as an invasive analysis, is challenging especially in confronting untreated biological samples. This deteriorates by the poor sensitivity and selectivity of methods to recognize cancer-linked proteins on-demand. Existing gold standard enzyme-linked immunosorbent assay (ELISA) and polymerase chain reaction (PCR) methods require enzymatic reactions and extra enhancements making them expensive and hard to operate. In recent years, biosensors have emerged as high-quality recognition tools for the detection of different diseases like cancer^9–12^. Considering the positive effect of nanomaterials in daily life and also in diagnostics^2,13^, numerous reports have been proposed nanomaterials enhanced biosensing strategies for the determination of cancer biomarkers^14–16^. Shiddiky et al. developed a sandwich-type immunosensor for the detection of breast cancer biomarkers (EpCAM). In this study, an ITO electrode was initially modified with anti-EpCAM antibodies (Ab1). After attachment of EpCAM protein biomarkers to the ntibody modified electrode, the as prepared graphene oxide- CdSe quantum dots (CdSe QDs) was drop casted onto the electrode as the reporter motif. The electrochemical signals were produced by the oxidation of CdSe QDs. Using this strategy, a limit of detection of 100 fg/mL was obtained^17^. In another study by Arkan and coworkers, an electrochemical immunosensor based on gold nanoparticles combined with multiwall carbon nanotube-ionic liquid (AuNPs/MW-CILE) was used for the detection of HER-2 protein. In this research, AuNPs were electrodeposited onto the MW-CILE modified working electrode. After that, the second layer of colloidal AuNPs was attached to the modified electrode via 1,6-hexanedithiol chemistry. Using 1-ethyl-3- (3-dimethylami-nopropyl) carbodiimide (EDC)/ N-hydroxysuccinimide (NHS) coupling chemistry, antibody molecules were attached to the modified electrode for the next determination of HER-2 concentrations down to 7.4 ng/mL^18^. Azahar Ali et. al developed an immunosensing protocol based on the graphene foam/titanium oxide nanofibers nanocomposites for the detection of epidermal growth factor receptor-2 (EGFR-2). Using EDC/NHS strategy, the antibodies were covalently bonded to the carboxylic groups of the nano-composite. The fabricated framework enabled to detection of EGFR-2 concentration with a high sensitivity of 0.585 μA/μM/cm^2^ and an acceptable LOD of 1 pM^19^. Shamsipur and colleagues used magnetic nanoparticles (Fe_3_O_4_ NPs) to develop a sandwich-like architecture for the quantification of HER-2 protein. Two components of the framework consisting of antibody functionalized Fe_3_O_4_ NPs as capture moiety modified onto the electrode and antibody functionalized AuNPs/Fe_3_O_4_ NPs composites as the reporter probe. After embedding HER-2 protein between the two components, silver was deposited onto the surface of AuNPs as the electrochemical indicator. In the presence of the target protein, the signals were increased. This approach could detect HER-2 as low as 20 fg/mL with a linear range of 50 ng/mL to 0.5 pg/mL (R^2^ = 0.9906)^6^.

Biocompatible polymers are frequently employed in a vast range of biomedical domains^20–22^. Poly(amino acid)s belong to the polyamides family forming only a single type of amino acid making them different from multi amino acid polyamides such as proteins. Among the poly(amino acid)s discovered to date, poly(glutamic acid) (PGA) has attracted the most attention due to itsnon-toxicity, water-solubility, excellent biocompatibility, and high functionability^23–26^. Owing to these features, PGA materials and composites have been promising in biomedical applications. Many reports have been published around the nanocomposites of PGA in various fields from tissue engineering to biosensors^27–29^. There have been many combinations of PGA with other nanomaterials in the literature used in biosensors^30–32^. One of the fascinating properties of GA is its capability of electrosynthesis in positive potentials (about 2 V), making it a good candidate for in situ electropolymerizations on the electrode transducers for high-quality polymeric films^31,33,34^. On the other hand, WO_3_ nanostructures have been broadly investigated in sensing applications owning to their chemical stability, acceptable biocompatibility, catalytic activity, and low cost^35,36^. However, there is little attention on WO_3_ based electrochemical biosensors for disease and cancer screening because of the relatively poor conductivity of synthesized nanostructures. The reason is the improper formation of WO_3_ nanoparticles leading to the unfavorable formation of nanomaterials onto the electrode. The deposition manner of nanomaterials onto electrodes plays a pivotal role in the stability, reliability, distribution, and conductivity of synthesized materials. Electrochemical grafting is a desirable method for *in-situ* deposition of nanostructures on electrode surfaces^37–40^. In this contribution, WO_3_ has the capability of electrodeposition from the acidic solution of its precursor compound (Na_4_WO_3_)^41^. For this purpose, the deposition solution should be highly acidic (pH ~ 1.4) because of the nature of the deposition reaction. On the other hand, the precursor possesses low solubility in acidic media (pH ~ 1.4), for which, H_2_O_2_ is added into the solution as a co-reactant to produce a complex with WO_3_ and making it highly soluble^42,43^. In this regard, discovering new protocols for the synthesis of WO_3_ nanostructures in mild conditions and with high conductivity can address the present challenges.

Therefore, the combination of biopolymers with WO_3_ nanostructures could be useful in designing biosensing platforms.

Commensurate with these descriptions^44,45^, in this research, we used the green co-electrosynthesis route to in situ generation of WO_3_/PGA nano-biocomposite. Unlike previous methods, no H_2_O_2_ was used in the synthesis of the nanocomposite. We employ a wide potential range (− 1 to 2.5 V) to simultaneously electrosynthesis of WO_3_ and PGA on the electrode. To the best of our knowledge, this is the first report on simultaneous deposition of WO_3_ and PGA. Also, to the best of our knowledge, this is the first time employing WO_3_/PGA combination for biosensing purposes.

## Experimental

### Material

HER-2 antibody (Ab) and HER-2 protein were obtained from Abcam. Na_2_WO_4_ powder was purchased from Merck. The glutamic acid powder was obtained from Sigma. 1-ethyl-3- (3-dimethylami-nopropyl) carbodiimide (EDC) and N-hydroxysuccinimide (NHS) solutions were prepared by dissolving their powders in deionized water, which were bought from sigma. Phosphate buffer saline (PBS, pH = 7.4) was prepared by adding KCl (200 mg), NaCl (8 g), Na_2_HPO_4_ (1.44 g), KH_2_PO_4_ (245 mg), into the deionized water at room temperature. Sulfuric acid and nitric acid solutions were prepared by dilution of a concentrated solution them using deionized water.

### Apparatus

Electrochemical measurements were preceded by applying a Metrohm Autolab controlling by Nova software. The electrochemical system concluded a three-electrode system consisting of a working electrode (glassy carbon electrode GCE with 3-mm in diameter), a counter electrode (Pt wire), and a reference electrode (Ag/AgCl). All the experimental performances including preparations, depositions, and measurements, proceeded in ambient conditions. To homogenize the solutions, an ultrasonic bath (Transsonic, model 420) was employed. The pH of solutions was measured by a pH meter (Corning, model 120). A magnetic stirrer model Heidolph was applied for the blending of solutions. The scanning electron microscope (SEM), Energy-dispersive X-ray spectroscopy (EDX), and dot blotting imaging experiments were implemented on Tescan, model MIRA3.

### Electrode preparation steps

Before preparation, the glassy carbon electrode (GCE) was polished physically and electrochemically using alumina powder and PBS solution (pH = 7.4) followed by washing with ultrapure water. Afterward, a precursor solution was manufactured by dissolving 1 g Na_2_WO_4_ powder and 0.11 g glutamic acid powder into the 10 mL PBS (pH 7.4). After sonication for 30 min, the pH of the solution was adjusted to 7.4. The WO_3_/p-Glu nanocomposite was then electrochemically synthesized using a three-electrode system at ambient temperature. The potential of the working electrode was scanned between − 1 and 2.5 V (0.1 V s^−1^) vs. reference electrode. As a result, H^+^ ions required for electrografting of WO_3_ were produced by applying high positive potentials (up to 2.5 V) resulting in a desirable high current of about 0.001 A. As described by previous studies, p-Glu and WO_3_ nanostructures were generated around 2 V^34,46^ and − 0.6 V^47,48^, respectively.

**Figure 1:**
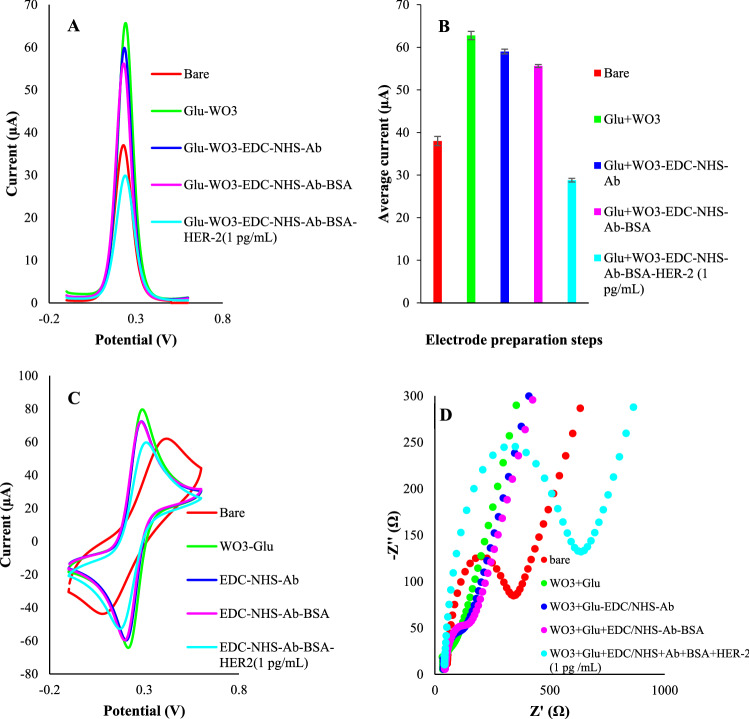
Different steps of electrode preparation. (**A**) the DPV voltammograms and (**B**) correlated histograms for each preparation step. (**C**) CVs and (**D**) impedance responses of different modification steps. The electrochemical measurements were obtained in a PBS buffer (pH = 7.4) of 5 mM K_4_[Fe(CN)_6_] and 0.1 M KCl. All the DPV measurements were obtained in tha potential range of − 0.1 to 0.6 V with pulse amplitude of 5 mV with interval time of 0.5 s.

After electrodeposition, a pre-prepared solution of EDC and Ab (18 µg mL^−1^) (1:1 v/v) was mixed with an NHS solution (2:1 v/v) and rest for 30 min. This activates the -COOH groups of Abs. In the next, 10 µL of EDC/NHS-Ab (18 µg mL) was incubated onto the modified electrode for 120 min (at 4 °C). After washing in 10 mM PBS solution (pH = 7.4), a droplet of 10 µL solution of HER-2 protein (in different concentrations) was incubated on the electrode for 3 h (at room temperature). This led to the formation of GCE-WO_3_/p-Glu-Ab-HER-2 on the electrode. The prepared electrode was carried out into an electrochemical cell in which the electrochemical measurements were proceeded using K_4_ [Fe (CN) _6_] solution as a redox agent. Both DPV and CV voltammograms were obtained in the potential range of − 0.1 to 0.5 V (0.1 V s^−1^). EIS experiments have been implemented for characterizing the sensor fabrication process. Nyquist plots consisted of two regions include a semicircle and a linear part that appeared at high frequencies low frequencies respectively. The diameter of the semicircle region (R_et_) reflects the charge transfer resistance. With creasing the diameter, the resistance was increased. Figure 1 represented the DPVs, CVs, and EIS results of the consecutive preparation stages of the proposed platform. As can be seen, after deposition of the PGA/WO_3_ platform, the DPV and CV current peaks were increased while the R_et_ was decreased. This means the increased conductivity of the electrodeposited platform. As illustrated, after incubation of EDC/NHS, antibody, and target protein, the current peaks of DPV and CV were decreased and the R_et_ was increased. This indicated that all EDC/NHS, antibody and target protein decrease the conductivity of the electrode surface. The equivalent circuit data corresponding to the Nyquist plots for electrode preparation steps were prepared and inserted in Table S1. Also, the equivalent circuit was prepared and presented in Fig. S1.

Informed consent was obtained from all the participants included in the study.

### Ethics approval and consent to participate

All patients were asked to complete the informed consent. All procedures of this study were approved by the Local Ethics Committee of Tabriz University of Medical Sciences (IR.TBZMED.VCR.REC.1400.150). All procedures were done under the declaration of Helsinki.

## Results and discussion

### Co-electrodeposition of WO_3_/glutamic acid

Several important tips should be considered for the cyclic voltammetry (CV) behavior of the electrochemical solutions. To this purpose, the CVs of Glu, Na_2_WO_4_, Glu/NaWO_4,_ and water were obtained and compared to each other. The exact exploration of the CVs can help to interpret the events on the electrode surface and prove the deposition route of the proposed nanocomposites. Glutamic acid was electropolymerized into PGA in positive potentials during which, an oxidation process occurred resulting in a cation radical (GA^+^°). In the following, the formed cation radical initiates a sequence of addition reactions to the nucleophilic centers (–COO^−^) of glutamic acid molecules leading to the formation of α-L-PGA^33^. The overall reaction is represented as bellow:

1 $$ {\text{HOOC - CH}}_{2} {\text{ - CH}}_{2} {\text{ - CH}}\;\left( {{\text{NH}}_{2} } \right){\text{ - COOH}}\mathop{\longrightarrow}\limits^{{{\text{electrochemical}}\;{\text{oxidation}}}}\left( {{\text{HOOC - CH}}_{2} {\text{ - CH}}_{2} {\text{ - CH}}\;\left( {{\text{NH}}_{2} } \right){\text{ - COOH}}} \right)^{ + } $$

2 $$ \left( {{\text{HOOC - CH}}_{{2}} {\text{ - CH}}_{{2}} {\text{ - CH}}\;\left( {{\text{NH}}_{{2}} } \right){\text{ - COOH}}} \right)^{ + } \mathop{\longrightarrow}\limits^{{{\text{ectropolymerization}}}}\left[ {{\text{NH - }}\left( {{\text{CH - CH}}_{{2}} {\text{ - CH}}_{{2}} {\text{ - COOH}}} \right){\text{ - COO}}} \right]_{{\text{n}}} $$

On the other hand, according to the pieces of literature, WO_3_ can be converted to its hydrate form (H_x_WO_3_) and sub-stoichiometric (WO_3-y_) species in different potentials. Also, the previous studies proved that conversion of WO_3_ into H_x_WO_3_ and WO_3-y_ occurs on about − 0.1 and − 0.5 V vs. Ag/AgCl respectively^49–52^. The reduced forms of WO_3_ provide better conductivity^52^.

3 $$ {\text{2WO}}_{4}^{2 - } + {\text{4H}}_{{2}} {\text{O}}_{{2}} + {\text{2H}}^{ + } \to {\text{W}}_{{2}} {\text{O}}_{11}^{2 - } + {\text{5H}}_{{2}} {\text{O}} $$

4 $$ {\text{W}}_{{2}} {\text{O}}_{11}^{2 - } + {\text{2H}}^{ + } \to {\text{WO}}_{{3}} + {\text{2O}}_{{2}} + {\text{H}}_{{2}} {\text{O}} $$

5 $$ {\text{WO}}_{{3}} + {\text{xH}}^{ + } + {\text{xe}}^{ - } \to {\text{H}}_{{\text{x}}} {\text{WO}}_{{3}} $$

6 $$ {\text{WO}}_{{3}} + {\text{2 yH}}^{ + } + {\text{2ye}}^{ - } \to {\text{WO}}_{{{3} - {\text{y}}}} + {\text{yH}}_{{2}} {\text{O}} $$

One of the main drawbacks of previous methods for electrodeposition of WO_3_ nanomaterials is the application of H_2_O_2_ as a strong oxidant which is unhealthy hazardous material that can lead to environmental problems. Several methods have been established for in-situ production of H_2_O_2_ and avoiding the consumption of bulk H_2_O_2_ reagent^53–56^. Among them, electrosynthesis of H_2_O_2_ from the aqueous solution of carbonate ions is a straightforward and convenient synthesis route^57^. This mechanism proceeds as follows:

7 $${CO}_{3}^{2-}\to {CO}_{3}^{^\circ -}+{e}^{-}$$

8 $${2CO}_{3}^{^\circ -}\to {C}_{2}{O}_{6}^{2-}$$

9 $${C}_{2}{O}_{6}^{2-}+{H}_{2}O\to {H}_{2}{O}_{2}+2H{CO}_{3}^{-}$$

Aspirating with such a mechanism, phosphate ions can be oxidized electrochemically into peroxodiphosphate in high anodic potentials (about 2 V)^58,59^. The mechanism of this route is ascribed as below:

10 $$2{PO}_{4}^{3-}\to {{P}_{2}O}_{8}^{4-}+{2e}^{-}$$

Peroxodiphosphate ions are oxidative anions that can oxidize reductive molecules. The required H_2_O_2_ is produced from the following equation^60^. This reaction is accelerated by the pre-produced peroxodiphosphate anions.

11 $$ {\text{2H}}_{{2}} {\text{O}} \to {\text{2H}}^{ + } + {\text{H}}_{{2}} {\text{O}}_{{2}} + {\text{2e}}^{ - } ,\;\;\;{\text{E}}^{ \circ } = {1}.{776 }\;{\text{vs }}\;{\text{normal}}\;{\text{ hydrogen }}\;{\text{electrode}}\;\left( {{\text{NHE}}} \right) $$

**Figure 2:**
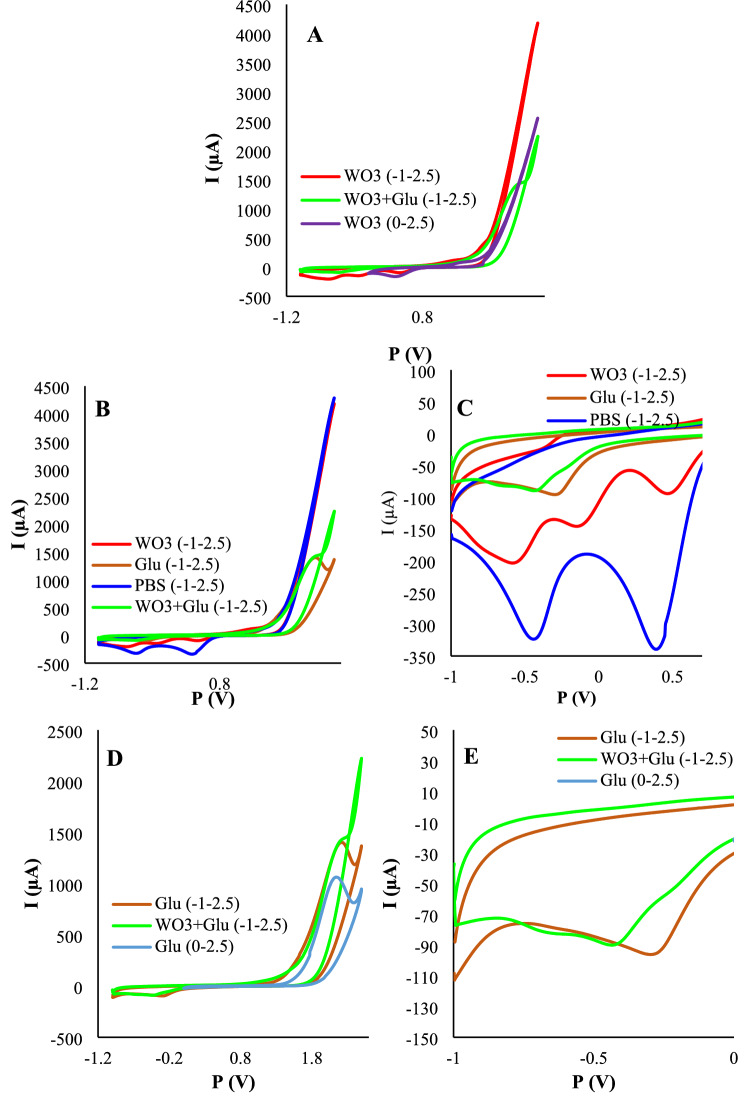
The electrochemical evaluation of the electrosynthesis methodology of WO_3_/p-Glu. (**A)** assessment of WO_x_ electrosynthesis in two different potential ranges (− 1 to 2.5 V and 0–2.5 V) and precursor compositions; (**B**) representative illustration of the composition effect of precursor on the shape, position, and intensity of the electrochemical peaks (all measurements proceeded in that range of – 1 to 2.5 V); (**C**) magnified image of panel (**B**); (**D**) investigation of p-Glu electrosynthesis in two different potential ranges (− 1 to 2.5 V and 0–2.5 V) and precursor compositions (glutamic acid, WO_3_/glutamic acid); (**E**) magnified image of panel (**D**). All the solutions were prepared in PBS (0.1 M, pH = 7.4). All the measurements were progressed in the scan rate of 0.1 V/s. The electrochemical measurements were obtained in a PBS buffer (pH = 7.4) of 5 mM K_4_[Fe(CN)_6_] and 0.1 M KCl.

Aspired by these mentioned reactions, we prepared four solutions of PBS, WO_3_/PBS, Glu/PBS, and WO_3_/Glu/PBS and applied different potential ranges on the GCE dipped into the solutions. The behavior of the solutions to the applied potential ranges discovered many interesting signs of WO_3_ and p-Glu electrodeposition (Fig. 2). According to the NaWO_4_/PBS CV, there are two peaks on about − 0.15 and − 0.7 V vs. Ag/AgCl which can be associated with Eqs. (3 and 4). With a reduced intensity, these two peaks were shown in Glu/NaWO_4_/PBS solution. These two mentioned peaks are absent in the PBS solution. In total, by using a broad potential range (− 1 to 2.5 V), both PGA and WO_3_ species were deposited onto the electrode at the same time. There is an important point in this case that is, the produced reduced forms during the negative cycle can turn into their oxidized forms during the next anodic cycle. There are two tips in this view. First, these consecutive reduction–oxidation processes produce a unity film instead of a layer-by-layer film which could increase the stability of the film. Second, the oxidized products were reduced again in the next cathodic cycle. Because the concentration of oxidized forms (WO_3_ and PGA^+^) increased from one step to the next step, the thickness of the deposited film is not constant and increases after each step. The CVs of 10 consecutive electrodeposition cycles were illustrated in Fig. S7 A-C. As can be seen, the deposition currents were increased from one step to the next one, which represented the more electrodeposited amount of the nano-biocomposite. Also, as represented, the increase rate was smoothened from one step to the next step. This means that the effect of the number of deposition cycles on film growth is limited by the number of cycles.

### Characterization

#### Electrochemical characterization

The effect of each modification step was investigated through CV, electrochemical impedance spectroscopy (EIS), and differential pulse voltammetry (DPV), techniques (Fig. 2). Also, the effect of the swept potential range was studied considering the charge transferring ability alterations. All the electrochemical measurements were obtained in a PBS solution (pH = 7.4) of 5 mM K_4_ [Fe(CN)_6_] and 0.1 M KCl. The DPV and CV experiments were taken placed in the range of 0.1 to 0.5 V (scan rate = 0.1 V/s). Moreover, EIS experiments have been implemented for characterizing the sensor fabrication process. Nyquist plots consisted of two regions include a semicircle and a linear part that appeared at high frequencies low frequencies respectively. The diameter of the semicircle region reflects the charge transfer resistance. With creasing the diameter, the resistance was increased. The electron transferring capability of the electrode surface and diffusion rate of redox agents were screened from the semicircle part and linear portion of EIS plots. These characteristics were individually obtained from peak heights of DPV curves.

**Figure 3:**
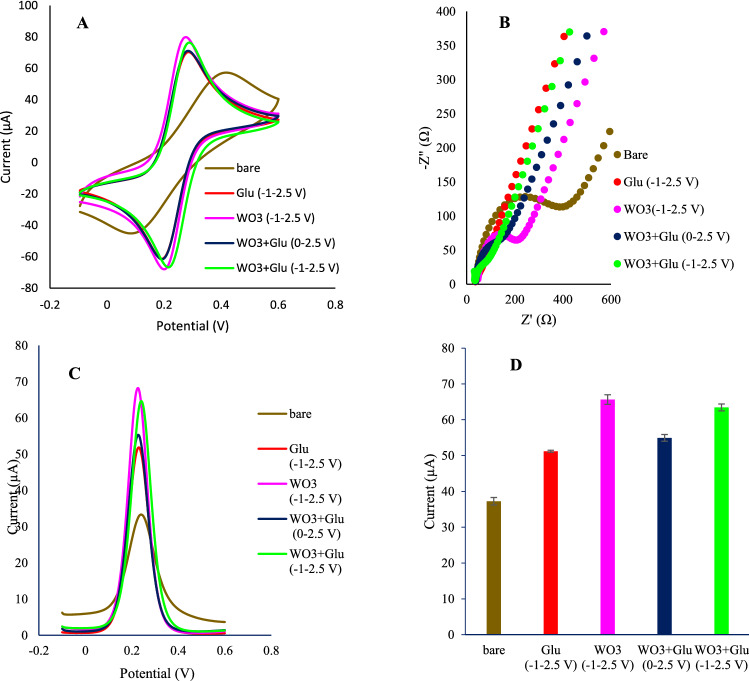
The effect of the potential range and composition of the electrodeposition solutions. (**A**) the CVs voltammograms; (**B**) EIS plots; (**C**) DPV voltammograms and (**D**) DPV correlated histograms. The electrochemical measurements were obtained in a PBS buffer (pH = 7.4) of 5 mM K_4_[Fe(CN)_6_] and 0.1 M KCl. All the DPV measurements were obtained in tha potential range of − 0.1 to 0.6 V with pulse amplitude of 5 mV with interval time of 0.5 s.

Following the modification of the electrode with WO_3_/p-Glu, the charge transferring resistance was dramatically changed. To study the effect of p-Glu and WO_3_ on conductivity, the electrode was separately modified with p-Glu and WO_3_. According to the results, both WO_3_ and p-Glu boosted the charge transferring ability of the surface and we noted that the effect of WO_3_ was more than that of the p-Glu. As expected, the co-electrodeposition of WO_3_/p-Glu showed a slight increase in conductivity compared to the p-Glu. After incubation of EDC/NHS-Ab, the resistance was enhanced as a result of the steric hindrance. This can be interpreted as a successful attachment protocol of Ab onto the electrode surface. To probe the effect of the potential range, the same protocol was adopted for two potential ranges (0–2.5 V and – 1 to 2.5 V). The EIS results (Fig. 3B) represented that the resistance was enhanced when potential swept in the range of 0–2.5 V in comparison to the – 1 to 2.5 V range. In this range, two moieties are formed including WO_3_ nanostructures and p-Glu. But there is no formation of reduced forms of WO_3_ which are form in − 0.1 and − 0.7 V vs Ag/AgCl. Because the reduced forms of WO_3_ represent better conductivity than WO_3_, the reduction of current density can have correlated to the lack of reduced forms production. In other words, in this potential range, in the obtained WO_3_/p-Glu nanocomposite, the ratio of p-Glu is higher than WO_3_. We noted that resistance is lower than the p-Glu-GCE. This may be due to the co-participation of the tungsten along with the growth of p-Glu on the electrode surface. The associated CVs readouts were recorded for each notified step which was in line with EIS results (Fig. 3A). As a proof of principle, the best results were obtained for the – 1 to 2.5 V potential sweep range (Fig. 3C,D).

#### Morphology and roughness characterization

**Figure 4:**
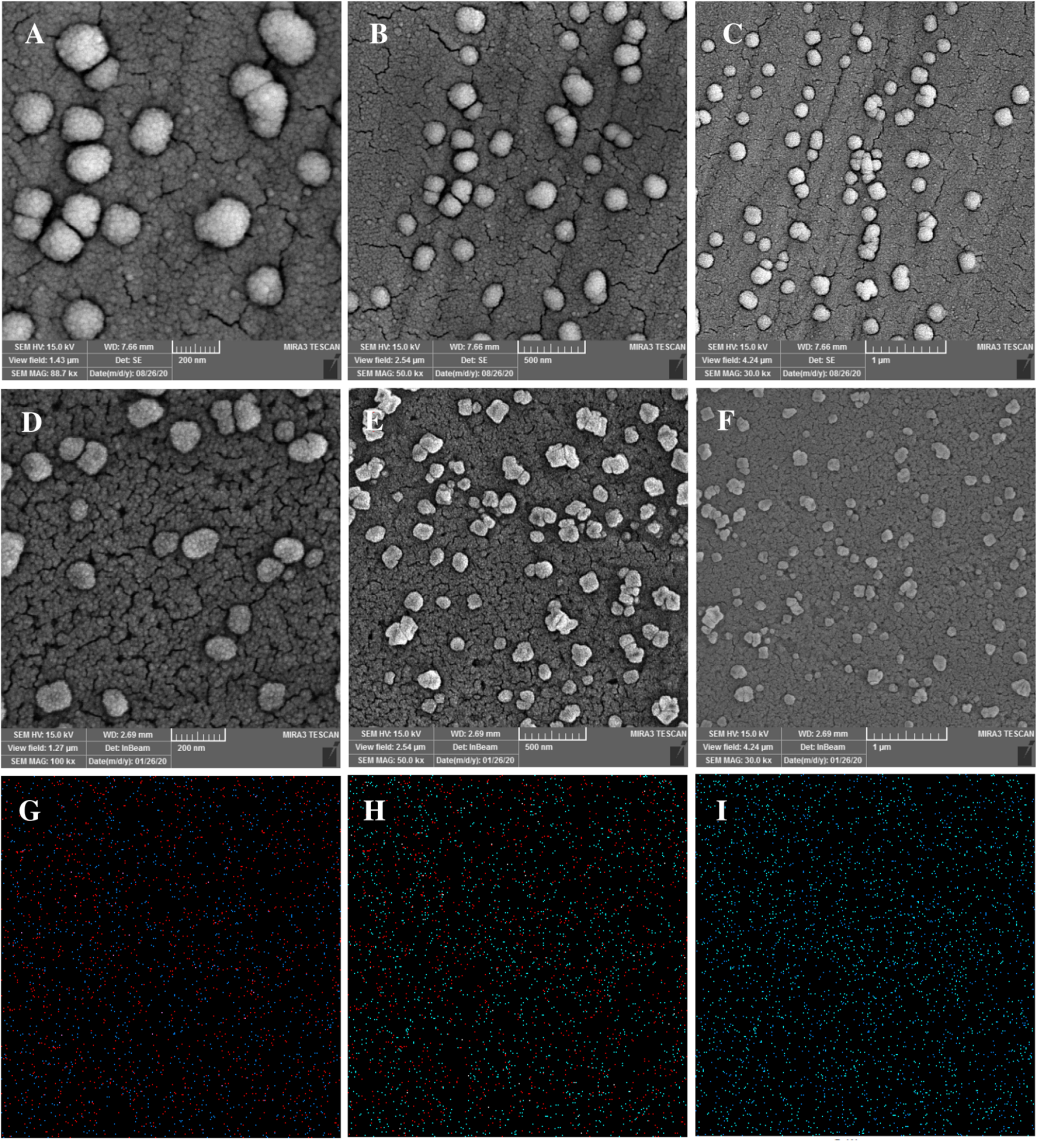
The SEM images of WO_3_/p-Glu (**A**–**C**), WO_3_/p-Glu-EDC-NHS-Ab (**D**–**F**) at different scales and dot mapping results of WO3/p-Glu (**G**–**I**).

To prove the electro-formation of p-Glu and WO_3_, morphology, lattice structure, and size distribution of the WO_3_ nanoparticles, scanning electron microscopy (SEM) imaging was employed. Also, the elemental analysis of the modified electrode surface was studied using Energy Dispersive X-ray spectroscopy (EDX). Figure 4 illustrated the surface modification quality of the electrode by WO_3_/p-Glu (− 1 to 2.5 V) nanoarchitectures. The SEM results presented a uniform, porous and high-quality electrodeposited nanocomposite on the electrode. For further confirmation, the dot mapping analysis was performed from the surface of the WO_3_/p-Glu modified electrode. As expected and shown in Fig. 4G–I, the W, O, and C atoms are uniformly distributed on the modified electrode surface with relatively close distance to each other. These relatively close distances between the W–O, W–C, and C–O correspond to the successful synthesis of WO_3_/p-Glu nanobiocomposite. The EDX results represented the appropriate electrodeposition of the WO_3_/p-Glu nanocomposites. The distribution quality of the electrodeposited nanocomposite was evidence by dot mapping photos. As shown in Fig. S2A and S2B, a satisfactory and homogeneously distribution manner was obtained.

### Optimization of the electrosynthesis step conditions

The number of cycles affects the electrochemical performance of the biosensor from two aspects via the thickness of the electrodeposited layers. First, the WO_3_ nanoparticles which cause the conductivity to be significantly boosted, and second, p-Glu which exerted a decreasing effect on charge transferring ability. On the other hand, the amount of p-Glu on the electrode plays a vital role in the amount of Abs which success to be covalently immobilized on the platform. In this regard, the different number of cycles (2, 5, 8, 12, 15, and 20) at the same scan rate (0.1 V s^−1^) and the same potential range (− 1 to 2.5 V), were investigated. The results illustrated in Fig. S3, as can be seen, the best results were obtained for 8 cycles. Further increase of cycle numbers has no obvious change on the signal outputs. By increasing the cycle number to 20, the electrochemical signals were decreased. This can be correlated to the alteration of the WO_3_/p-Glu ratio to be decreased.

### Analytical performance

**Figure 5:**
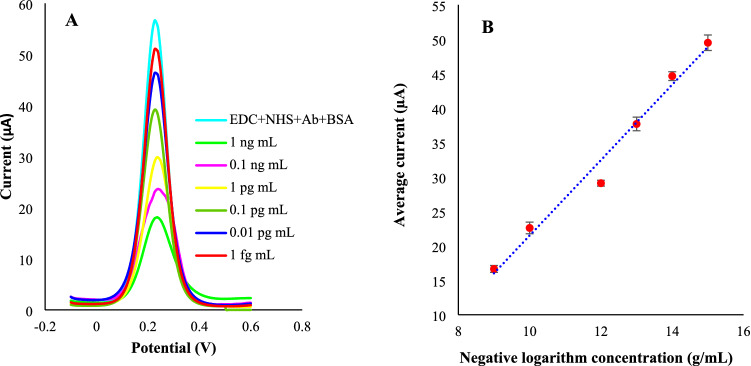
The concentration-electrochemical signals correlation using the proposed immunosensor. (**A**) the current–potential signals and (**B**) the obtained signals for different concentrations of the HER-2 protein (n = 2). The electrochemical measurements were obtained in a PBS buffer (pH = 7.4) of 5 mM K_4_[Fe(CN)_6_] and 0.1 M KCl. All the DPV measurements were obtained in tha potential range of -0.1–0.6 V with pulse amplitude of 5 mV with interval time of 0.5 s.

The DPV peak heights were obtained at different concentrations of HER-2 protein. As represented in Fig. 5, the signal readouts were declined by increasing HER-2 concentration. The peak heights presented good linearity with the logarithm of the concentration of HER-2 protein) (1 ng mL^−1^ to 1 fg mL^−1^).

The limit of detection (LOD) was gained to be 1 fg/mL. Compared to the formerly reported electrochemical^17,61,62^ for HER-2 determination, the proposed nanoimmunoassay possesses lower LOD and consequently better sensitivity. This high performance can be ascribed to the electrocatalytic activity and high conductivity of WO_3_ nanostructures.

To evaluate the reproducibility, the relative standard deviation (RSD%) was measured for 1 fg/mL of HER-2 protein using three different electrodes. The obtained RSDs indicated good reproducibility for both concentrations (3.42%). The results were presented in Fig. S4.

The signal stability of the prepared immunosensor was assessed via 10 consecutive DPV measurements. The obtained RSD for 10 consecutive DPV readouts represented good signal stability of about 1%. This can be obtained from two important features of the designed platform: (I) the rich functional groups of p-Glu and its strong binding to the Ab molecules; (II) highly ordered and stable electrodeposited WO_3_/p-Glu nanocomposite with high durability and strength. The obtained DPV voltammograms and correlated histograms are shown in Fig. S5.

To assess the specificity of the developed electrochemical immunosensor several possible interferences (carcinoma embryonic antigen (CEA), bovine serum albumin (BSA)) and the mixture of them in real samples were examined in a 100-fold concentration of HER-2 (Fig. S6). Weak electrochemical readouts were perceived in the presence of annoying species only. Whiles, an intense change in electrochemical responses were observed by adding HER-2. These observations proved the desirable selectivity of the proposed biosensor.

To give a lucid view of the proposed strategy, it was compared with several previously reported methods. The summary of the comparison was represented in Table 1. According to the evidence, the introduced framework possessed a desirable figure of merits in comparison with the other protocols.

**Table 1:** Comparison of the proposed biosensor with the other previously reported platforms for HER-2 protein.

Applied nanomaterial	LOD	LDR	Synthesis mechanism	Synthesis time of nanomaterials (min)	Refs.
Fe_3_O_4_-AuNPs-AgNPs	20 fg/mL	0.0005–50 ng/mL	Chemical coprecipitation	1710	^6^
Gr NSs CdSe QDs	100 fg/mL and 1 pg/mL in PBS and serum	0.0001–0.1 and 0.1–10,000 ng/mL	Wet chemical reaction	4500	^17^
Graphene foam TiO_2_ nanofibers	185 pg/mL	185 pg/mL–18.5 mg/mL for EIS 18.5 ng/L–18.5 mg/mL for DPV	Electrospinning	510	^63^
ZnO nanofibers	185 fg/mL	185 pg/mL–92 mg/mL	Electrospinning	1020	^64^
Fe_3_O_4_ NPs Au NPs	0.995 pg/mL	0.01–10 and 10–100 ng/mL	Wet chemical reaction Electrodeposition (CVs − 0.5 to 0.5 V)	280 20	^65^
Au NPs	0.01 ng/mL	0.01–100 ng/mL	Chemical reduction	~ 60	^66^
Au NPs	7.4 ng/mL	10–110 ng/mL	Electrodeposition (chronoamperometry − 0.4 V)	~ 6	^67^
WO_3_/p-Glu nanocomposites	1 fg/mL	1 ng/mL–1 fg/mL	Electrodeposition (CVs – 1 to 2.5 V)	~ 10	This work

There are some tips about the above-mentioned methodologies. Of course, we noted that each protocol has its advantages and restrictions and this deal is not to underestimate or minimize their qualities.

In the development of electrochemical biosensors using nanomaterials, two important tips should be notified. First, the biocompatibility and abundance of the ingredients (include nanoarchitectures) of the transducing framework are of great significance from the economical-environmental viewpoint. Some (nano) materials are high-performance but poor in biocompatibility like cadmium-based materials^68–70^. Some nanomaterials are of high biocompatibility but highly expensive and low abundance like platinum, gold, and silver nanomaterials One of the most important drawbacks of the Au nanomaterials is their high costs which prevent them to be employed in commercial and even, sometimes, experimental extent^71,72^. Some other (nano)materials represent desirable biocompatibility but with (very) low conductivity and then efficiency than the high conductive materials (such as Pt, Au, Ag) like several biopolymers (such as glutamic acid). On the other hand, some nanomaterials are of great interest for their conductivity and biocompatibility nut poor in functionability like WO_3_ nanostructures. The second tip is the preparation of the applied (nano)materials. Electrochemical synthesis strategies are promising ways of synthesis in which there is no need for extra reduction or other reagents which are most hazardous. Just a proper potential or current are implemented with no environmental issues. Considering these facts, the combination of biopolymers with WO_3_ nanostructures could be a response to the ask of new platforms with low costs and high biocompatibility. Regarding the presented experiments and the results which illustrated the proposed platform of p-Glu/WO_3_ nano-biocomposite as a high-performance platform for cancer screening the suggested strategy could address the present problems like biocompatible and final costs. As represented in Table 1, electrochemical synthesis routes possess a cost-effective methodology compared to the other procedures. In addition, the low volume consumed reagents is of great importance from commercial and environmental standpoints. A quick view of Table 1 can be a schematic comparison of different synthesis methods from several aspects.

### Clinical samples

**Figure 6:**
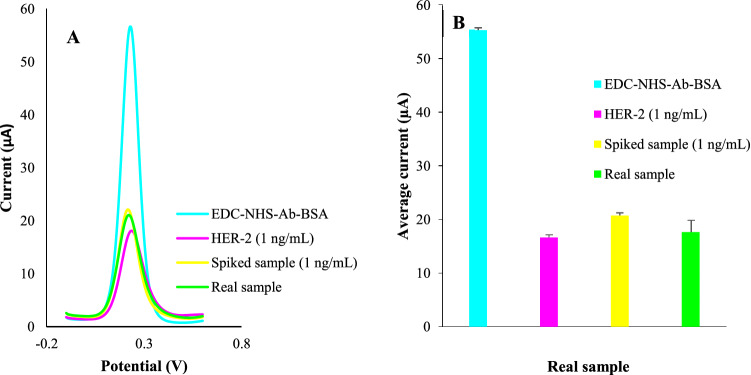
Analysis of a real sample of HER-2 positive patient and comparison of the correlated signals with a standard solution of HER-2 protein (1 ng/mL) and healthy real sample spiked with HER-2 protein (1 ng/mL). The electrochemical measurements were obtained in a PBS buffer (pH = 7.4) of 5 mM K_4_[Fe(CN)_6_] and 0.1 M KCl. All the DPV measurements were obtained in tha potential range of -0.1–0.6 V with pulse amplitude of 5 mV with interval time of 0.5 s.

To evaluate the applicability of the suggested strategy, it was implemented for untreated normal and patient samples. The normal sample was spiked with HER-2 protein (1 ng/mL) before analysis for matrix effect exploration (n = 2). The recovery results represented a matrix effect of 125% which is good considering the measurement in untreated serum matrix. Also, we tried an untreated HER-2 positive serum sample which indicated the competency of the proposed framework for biological and clinical point of care utilities. All the results were depicted in Fig. 6.

## Conclusions

WO_3_/p-Glu nanocomposite was synthesized through a biocompatible electrochemical process. To this end, the nanocomposite was synthesized by only dipping an electrode into the PBS solution containing Na_4_WO_4_ and glutamic acid. This process was followed by an appropriate potential sweep on the electrode. Compared to the previous methods developed for electrodeposition of WO_3_ nanostructures, which employed H_2_O_2_ as an extra co-reactant, no H_2_O_2_ was added to the precursor solution, but H_2_O_2_ was produced in situ using the water oxidation-splitting route. WO_3_ nanoparticles and p-Glu were prepared simultaneously in the sense that water acts as a precursor of H_2_O_2_, and phosphate (from PBS) and tungsten rolled as triggering agents for H_2_O_2_ production. Several morphological and electrochemical characterization analyses were used to prove the protocol. The results exhibited a high catalytic activity of the WO_3_ nanocomposite being as a biosensing platform. The implemented methodology can be appreciated from several points of view including biocompatibility and environmentally friendly nature, time and cost-effective properties, and high performance in the screening of breast cancer incidence.

## Supplementary Information

Supplementary Information.
