# Supplementary material for: Application of green synthesized WO3-poly glutamic acid nanobiocomposite for early stage biosensing of breast cancer using electrochemical approach
Source: Sci Rep. 2021 Dec 14;11:23994. doi: 10.1038/s41598-021-03209-8 (PMC8671486; doi:10.1038/s41598-021-03209-8)

**Application of green synthesized WO_3_-poly glutamic acid nanobiocomposite for early stage biosensing of breast cancer using electrochemical approach**

Hassan Nasrollahpour^a^, Abdolhossein Naseri^a*^, Mohammad-Reza Rashidi^b^, Balal Khalilzadeh^c*^

^a^ Department of Analytical Chemistry, Faculty of Chemistry, University of Tabriz, Tabriz, Iran.

^b^ Research Center for Pharmaceutical Nanotechnology (RCPN), Tabriz University of Medical Sciences, Tabriz, Iran.

^c^ Stem Cell Research Center, Tabriz University of Medical Sciences, Tabriz, Iran.

**Corresponding authors:**

**Balal Khalilzadeh***, PhD, Stem Cell Research Center (SCRC), Tabriz University of Medical Sciences, 51664-14766 Tabriz- Iran, Tel: +98(41)-33363311; Fax: +98(41)33363231*

*Email: khalilzadehb@tbzmed.ac.ir,* [*balalkhalilzadeh@gmail.com*](mailto:balalkhalilzadeh@gmail.com)

**Abdolhosein Naseri**, PhD, Department of Analytical Chemistry, Faculty of Chemistry, University of Tabriz, PO Box 51644‐14766, Tabriz, Iran. Email: [a_naseri@tabrizu.ac.ir](mailto:a_naseri@tabrizu.ac.ir)

**Figure and table captions**

**Table S1:** Equivalent circuit data for different electrode preparation steps and Equivalent circuit.

**Figure S1:** EDX and quantitative elemental analysis of WO_3_/p-Glu (A) and WO_3_/p-Glu-EDC-NHS-Ab (B).

**Figure S2:** The effect of the number of deposition cycles on the signal outputs of the proposed platform. A) the DPV responses and B) the correlated histograms. All the DPV measurements were obtained in tha potential range of -0.1-0.6 V with pulse amplitude of 5 mV with interval time of 0.5 s.

**Figure S3:** A) The DPV voltammograms obtained in the same measurement conditions (PBS, pH 7.4, K_4_Fe(CN)_6_/K_3_Fe(CN)_6_ 5 mM) for three different electrodes which were prepared similarly. B) the correlated histograms. All the DPV measurements were obtained in tha potential range of -0.1-0.6 V with pulse amplitude of 5 mV with interval time of 0.5 s.

**Figure S4:** The signal stability of the proposed biosensor obtained for 0.01 pg/mL HER-2 protein. A) the obtained 10 consecutive DPV voltammograms and B) the correlated histograms. All the DPV measurements were obtained in tha potential range of -0.1-0.6 V with pulse amplitude of 5 mV with interval time of 0.5 s.

**Figure S5:** The selectivity of the platform for HER-2 protein against two different possibly interferences (CEA and BSA) (n=2). All the DPV measurements were obtained in tha potential range of -0.1-0.6 V with pulse amplitude of 5 mV with interval time of 0.5 s.

Figure S6: The investigation of the effect of each cycle on the electrodeposition of the platform. CV voltammograms between, A) -1 to 2.5 V, B) -1 to 0 V and C) 0-2.5 V.

**Figure S7:** Equivalent circuit of the electrode preparation steps.

**Table S1**

| **Modification steps** | **R_s_** | **C_dl_** | **R_ct_** | **W1-R** | **W1-T** | **W1-P** |
| --- | --- | --- | --- | --- | --- | --- |
| **Bare** | 54.69 | 1.11E-06 | 224.6 | 1.01E+07 | 1.57E+09 | 0.43032 |
| **+WO_3_+Glu** | 35.43 | 4.01E-06 | 38.11 | 4.49E+06 | 2.20E+07 | 0.49584 |
| **+EDC-NHS+Ab** | 38.48 | 3.43E-06 | 64.41 | 1.08E+07 | 2.13E+08 | 0.48137 |
| **+EDC-NHS+Ab+BSA** | 45.08 | 3.63E-06 | 77.78 | 1.23E+08 | 3.20E+10 | 0.48286 |
| **+EDC-NHS+Ab+BSA+Ag** | 43.74 | 1.03E-06 | 458.3 | 3.91E+07 | 5.42E+10 | 0.4223 |

**Figure S1**


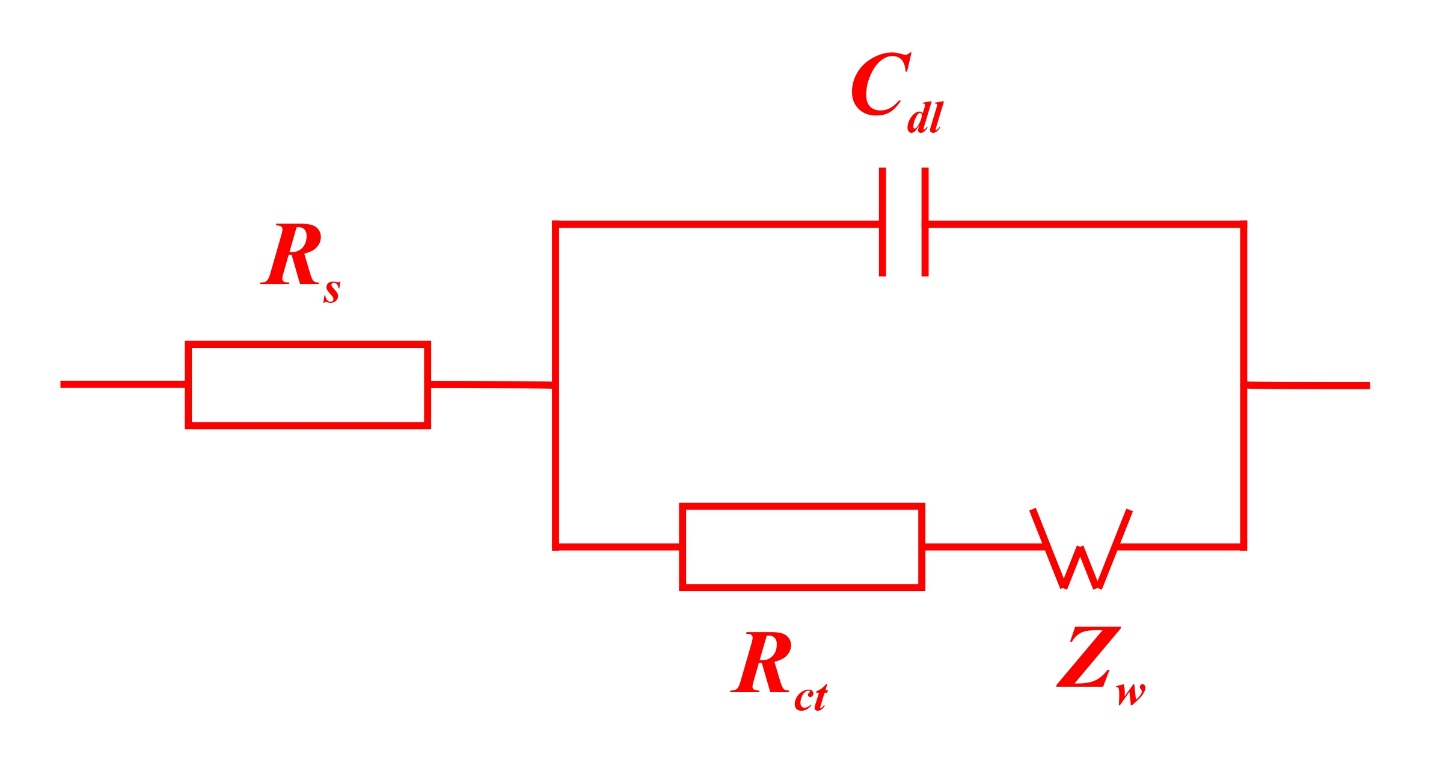


**Figure S2A:**

**A**

| Element | C | N | O | Na | W |  |
| --- | --- | --- | --- | --- | --- | --- |
| W% | 33.28 | 18.25 | 8.48 | 4.46 | 35.53 | 100 |

**Figure S2B:**

**B**

| Element | C | N | O | W |  |
| --- | --- | --- | --- | --- | --- |
| W% | 67.92 | 12.76 | 9.52 | 9.80 | 100 |

**Figure S3:**


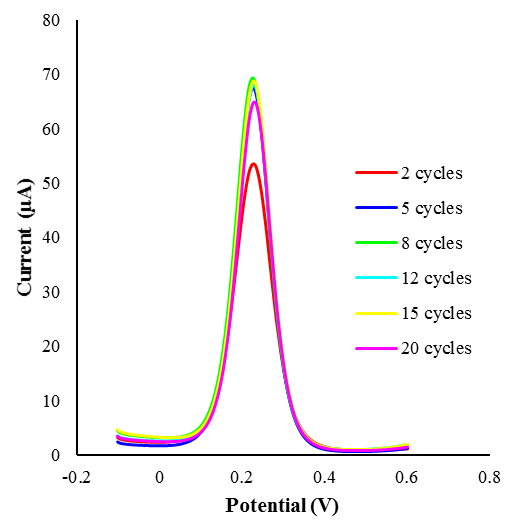


**A-0.0004**


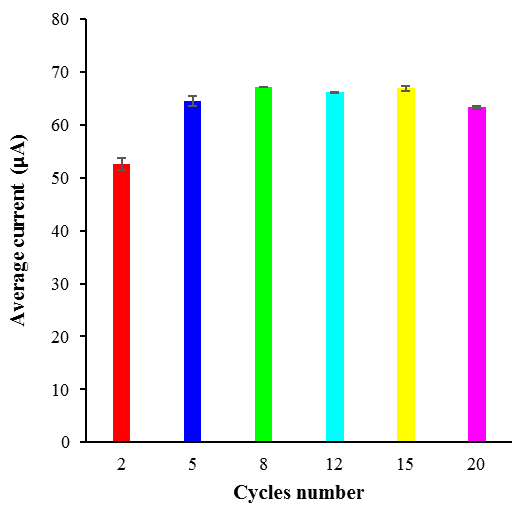


**B**

**Figure S4:**


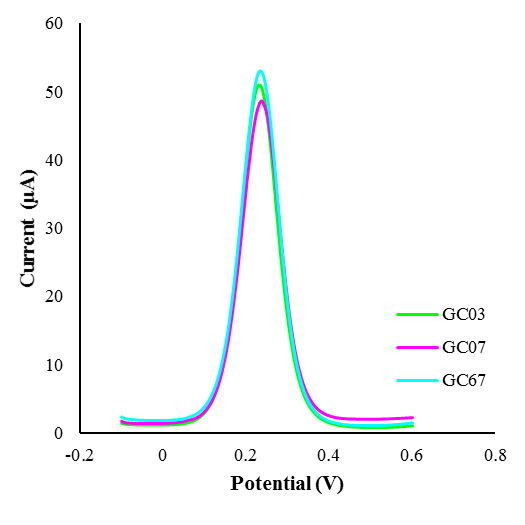


**A-0.0004**


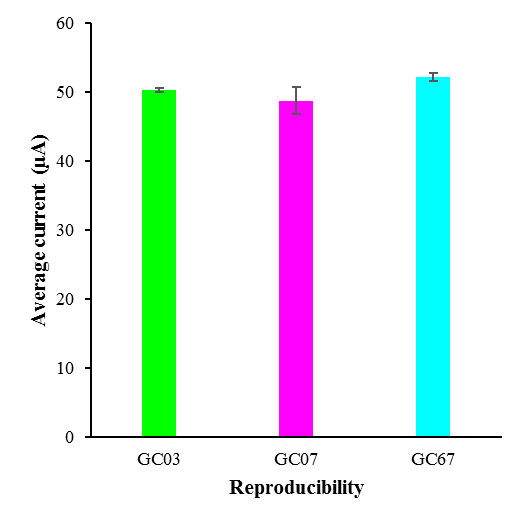


**B**

**Figure S5:**


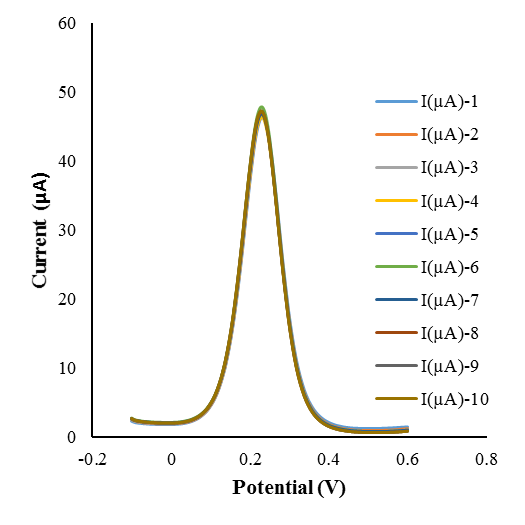


**A-0.0004**


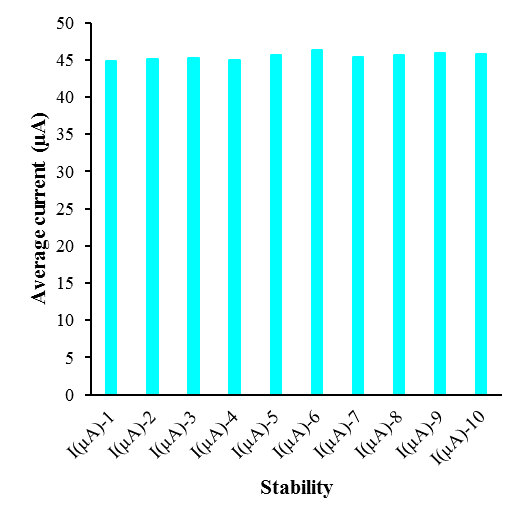


**B**

**Figure S6:**

**Figure** **S7:**


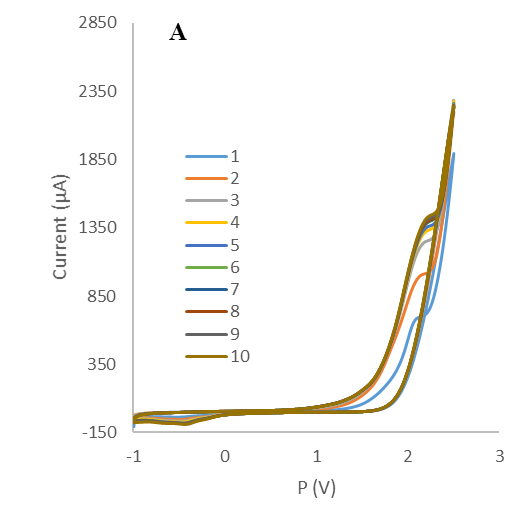

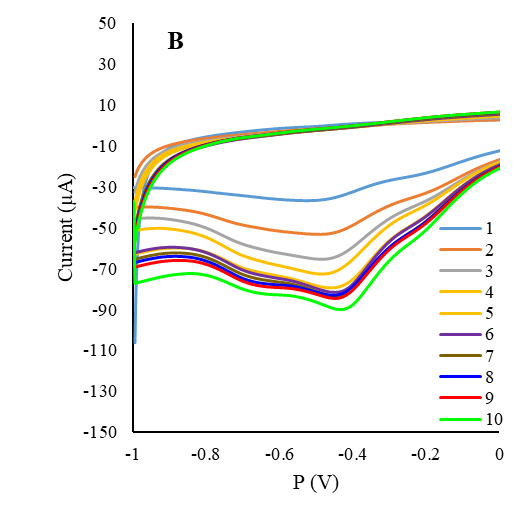

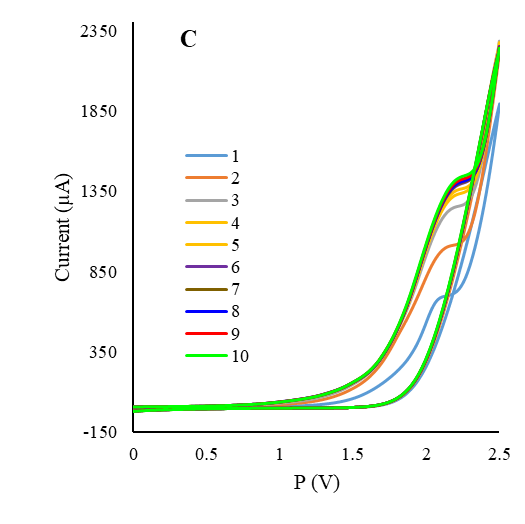

Supplement: Supplementary file 1 — Supplementary Information. [file 41598_2021_3209_MOESM1_ESM.docx]
